# *Streptococcus suis cps7*: an emerging virulent sequence type (ST29) shows a distinct, IgM-determined pattern of bacterial survival in blood of piglets during the early adaptive immune response after weaning

**DOI:** 10.1186/s13567-018-0544-8

**Published:** 2018-06-15

**Authors:** Karoline Rieckmann, Anna Seydel, Kristin Szewczyk, Kerstin Klimke, Viktoria Rungelrath, Christoph Georg Baums

**Affiliations:** 1 Institute for Bacteriology and Mycology, Centre for Infectious Diseases, Faculty of Veterinary Medicine, University Leipzig, 04103 Leipzig, Germany; 2 Institute for Veterinary Pathology, Faculty of Veterinary Medicine, University Leipzig, 04103 Leipzig, Germany

## Abstract

*Streptococcus (S.) suis* is an important porcine pathogen causing meningitis, arthritis and septicemia. As *cps7* emerged recently in Germany in association with severe herd problems, the objective of this study was to characterize the geno- and phenotype of invasive *cps7* strains. Twenty *cps7* strains were isolated from diseased pigs from different farms with *S. suis* herd problems due to meningitis and other pathologies. Eighteen of the *cps7* isolates belonged to sequence type (ST) 29. Most of these *cps7* strains secreted a short MRP variant in agreement with a premature stop codon. Expression of Ide_*Ssuis*_, an IgM specific protease, was variable in four further investigated *cps7* ST29 isolates. Bactericidal assays revealed very high survival factors of these four *cps7* ST29 strains in the blood of weaning piglets. In growing piglets, the increase of specific IgM led to efficient killing of *cps7* ST29 as shown by addition of the IgM protease Ide_*Ssuis*_. Finally, virulence of a *cps7* ST29 strain was confirmed in experimental infection of weaning piglets leading to meningitis and arthritis. In conclusion, this study characterizes *cps7* ST29 as a distinct *S. suis* pathotype showing high survival factors in porcine blood after weaning, but IgM-mediated killing in the blood of older growing piglets. This underlines the relevance of IgM as an important host defense mechanism against *S. suis*.

**Electronic supplementary material:**

The online version of this article (10.1186/s13567-018-0544-8) contains supplementary material, which is available to authorized users.

## Introduction

*Streptococcus suis* is one of the most important porcine pathogens and an emerging human pathogen. It causes meningitis, arthritis and septicemia in piglets, mainly at an age of 4–10 weeks, leading to major economic losses [1]. It is one of the main reasons for antimicrobial use in piglets. The diversity of this bacterial species with at least 29 serotypes [2–7] makes it a rather challenging pathogen with regard to vaccine development. There are not only differences between the serotypes but also within one serotype regarding virulence-associated factors, antimicrobial susceptibilities and clonal complexes [8, 9]. So far 960 *S. suis* sequence types have been added to the MLST database available at http://www.pubmlst.org. Generally, a clonal complex is dominated by one serotype (*cps*) [10, 11]. Worldwide *cps2*, *9*, *3*, *1/2* and *7* are most important with *cps2* being the predominant one in North America (almost equal to *cps3* with 24.3 and 21%, respectively), South America and Asia whereas in Europe, based on data from Spain and the Netherlands from 2002 to 2013, it is presently *cps9*, followed by *cps2* and *cps7* [6, 12]. Before the year 2000, *S. suis cps1* was prevalent in Belgium and the United Kingdom and *S. suis cps7* was frequently isolated in Denmark during the 1990s, but recent data from these and many other European countries are lacking [6, 12, 13]. In *S. suis cps2*, a muramidase-released protein (MRP) and extracellular protein factor (EF) positive phenotype as well as expression of suilysin are associated with virulence [12, 14, 15]. However, an *epf*+ genotype has rarely and an EF+ phenotype has never been described for *cps7* [12, 16]. MRP enables the bacteria to bind to human fibrinogen and this interaction increases migration of *S.* *sui*s across a human cerebral microvascular endothelial cell barrier [17, 18]. Through PCR, at least six *mrp* variants can be differentiated based on the size of the amplicon, of which mrp* and *mrp*^S^ are the most frequent ones [19]. Different serotypes express the immunoglobulin M-degrading enzyme of *S. suis* (Ide_*Ssuis*_) [20]. This protease is highly specific as it cleaves only porcine IgM but not IgG or IgA. Furthermore, changes in band patterns of host proteins present in different body liquids elicited by addition of rIde_*Ssuis*_, can all be explained by IgM cleavage [20]. Ide_*Ssuis*_ cleaves porcine IgM between the C2 and C3 domain. This is thought to be a complement evasion mechanism as the putative C1q binding motif is located in the C3 domain [21]. Nevertheless none of the virulence-associated factors have been proven to be essential for infection in pigs [12].

As placentation in pigs is epitheliochorial, new born piglets are dependent on colostrum for uptake of maternal immunoglobulins (Ig). IgG represents the main Ig fraction with more than 80%, whereas IgA and IgM levels in colostrum are lower with ~13 and ~4%, respectively [22]. This leads to high immunoglobulin levels in the piglets’ sera with a peak at 24 h after birth. Ig levels then decrease in sow milk as well as in the piglets’ serum resulting in minimum levels depending on the Ig type. The lowest level of IgM is reached at 8–14 days whereas IgG levels are lowest at 36–40 days and IgA levels at 17-22 days [22]. In this study we follow up survival of *S. suis* serotype 7 (*cps7*) in the blood of weaning and growing pigs and show restriction of survival by increase of specific IgM.

## Materials and methods

### Bacterial strains and growth conditions

Twenty-two *cps7 S. suis* isolates from pigs from herds in Germany (*n* = 18) and Austria (*n* = 2) were included in this study (Table 1). All but two originated from piglets with acute clinical signs, these two were isolated from healthy carrier pigs. *S. suis* strains D282 (*mrp*+ *cps*2), A3286/94 (mrp* *cps*9), 90-2741-7 (mrp*** *cps2*), V7353/1 (mrp**** *cps*7) and T15 (*mrp* negative *cps*2) were included for PCR differentiation of *mrp* variants [19, 23]. *S. suis* strain 10 is an MRP+ EF+ Ide_*Ssuis*_+ suilysin+ *S. suis cps2* strain used to generate the isogenic *cps*EF, *mrp* and ide_*Ssuis*_ mutants 10cpsΔEF, 10M7 and 10Δide_*Ssuis*_, respectively [20, 24, 25]. *S. suis* 16085/3b is a recent *mrp*+ *sly*+ *cps9* isolate from the spleen of a herd with a substantial *cps9* herd problem due to septicemia and meningitis. Bacteria were grown either on Columbia agar plates with 6% sheep blood (Oxoid, Wesel, Germany) or in Bacto™ Todd Hewitt Broth (THB) at 37 °C for 24 h or overnight, if not stated otherwise. The bacterial species was verified by MALDI-TOF MS analysis using Biotyper Microflex LT (Bruker Daltonik GmbH, Bremen, Germany) as recommended by the manufacturer.

**Table 1:** **Genotypic characterization and clinical background of** ***cps7 S. suis*** **strains investigated in this study**

Strain^a^	Genotype			Site of isolation	Year of isolation
	MP-PCR^b^	Sequence type	*mrp* variant^b^		
08/1324-1	*cps7/mrp*	29	*mrp*	Brain	2009
V2217/2	*cps7/mrp*	29	*mrp*	Lung	2010
S5552/1	*cps7/sly*	89	–	Brain	2010
V154/3	*cps7/mrp*	29	*mrp*	Brain	2012
V592/1	*cps7/mrp*	29	*mrp*	Lung	2012
R1984-1+/1	*cps7/mrp*	29	mrp****	Brain	2012
#451	*cps7/mrp*	29	mrp*	Brain	2012
13-00283-02	*cps7/mrp*	29	mrp****	Brain	2013
V3667/1	*cps7/mrp*	29	mrp*	Brain	2013
A2055/1	*cps7/mrp*	29	*mrp*	Joint	2013
V2310/1	*cps7/mrp*	29	*mrp*	Brain	2013
V3052/2	*cps7/mrp*	29	*mrp*	Lung	2013
D14412/3	*cps7/mrp*	29	mrp***	Brain	2014
15/2-7	*cps7/mrp*	29	*mrp*	Spleen	2015
16-00654-02	*cps7/mrp*	29	mrp****	Joint	2016
16-00654-03	*cps7/mrp*	29	mrp****	Pericardium	2016
16-00552-05	*cps7/mrp*	777	*mrp*	Brain	2016
16-00131-08	*cps7/mrp*	29	*mrp*	Brain	2016
16-00052-01	*cps7/mrp*	29	*mrp*	Unknown	2016
16-00596-02	*cps7/mrp*	29	mrp*	Brain	2016
30T^c^	*cps7*	nd	–	Tonsil	2014
262/3^c^	*cps7*	nd	–	Tonsil	2015

nd: not defined.

^a^All strains were isolated from piglets of different herds, except 16-00654-02 and 16-00653-03 as well as 30T and 262/3, respectively.

^b^All strains were genotyped in the multiplex (MP) PCR and in the *mrp* variant PCR described by Silva et al. [19]. None of the strains was positive for the *epf* gene encoding EF.

^c^Sequences of MLST alleles of strains 30T and 262/3 have also been submitted to the MLST database, but the sequence type has not yet been assigned (id 1792 and 1793, respectively).

### Genotyping

Screening of the *cps7* isolates by PCR including detection of the *cps7H* gene and differentiation of variants of the gene encoding the muramidase-released protein (*mrp*) was conducted as described previously [19], using lysates of *S. suis cps7* colonies grown on blood agar plates. For this, colony material was diluted in 100 µL deionized water and lysed at 270W in a microwave for 10 min. Two microliters of the lysate were used as template. *S. suis* strains D282 (*mrp*), A3286/94 (mrp*), 90-2741-7 (mrp***) and V7353/1 (mrp****) served as reference strains for the indicated *mrp* variants and T15 as negative control in this PCR [19]. Noteworthy, the size of the *mrp* amplification product generated in this PCR defines the *mrp* variant: 747 bp for *mrp*^S^, 1148 bp for *mrp*, 1556 bp for mrp*, 1600 bp for mrp**, 2000 bp for mrp*** and 2400 bp for mrp**** [19]. The *mrp* genes of four randomly selected *S. suis cps7* strains, which fulfilled the criteria to carry different *mrp* variants, were sequenced using Sanger Cycle Sequencing/Capillary Electrophoresis (for used primers see Additional file 1). Sequence data have been submitted to GenBank and are available under accession numbers MG214967 to MG214970.

Multi locus sequence typing (MLST) was performed as described, with published primers for the genes *gki, dpr, thrA, cpn60, recA* [10], *mutS* [23] and new primers for the gene *aroA* (aroA_KSvH_rev: AATTCGCTACCAACTCCCTG, aroA_KSvH_for: AAGGTAATAATCGGCAACTC).

### Western blot analysis

Culture and protoplast supernatants were obtained of all investigated *cps7* strains shown to carry *mrp* by multiplex PCR. The *cps2* strain 10 and its isogenic *mrp* and *ide*_*Ssuis*_ mutants 10M7 and 10Δide_*Ssuis*_, respectively, served as controls as indicated [20, 24]. Following 30-fold concentration of the culture supernatants with Amicon Ultra 15-mL centrifugal filters with a 30-kDa cutoff (Merck Millipore, Darmstadt, Germany), samples were prepared with reducing sample buffer and separated in separating and stacking gels containing 10 and 4% acrylamide, respectively. After blotting to a nitrocellulose membrane (Roti^®^-NC HP 40.1, Roth, Karlsruhe, Germany) and blocking with 5% skimmed milk powder dissolved in Tris-buffered saline-Tween 20 (TBST) the primary polyclonal antibody was applied in 1% skimmed milk TBST and at a 1:1000 dilution (rabbit-anti-MRP or rabbit-anti-Ide_*Ssuis*_ as indicated) [20, 26]. Blots were washed 4 times with TBST. A goat-anti-rabbit polyclonal horseradish peroxidase—(HRP) conjugated antibody (Dianova, Hamburg, Germany) served as secondary antibody (1:50 000 in 1% skimmed milk TBST). The Western blot was developed using SuperSignal™ West Pico PLUS Chemiluminescent Substrate (Thermo Scientific, Schwerte, Germany) as recommended by the manufacturer. Additionally, anti-porcine-IgM Western blots were carried out as described above to detect IgM cleavage products in plasma after bactericidal assays. A goat-anti-porcine-IgM (Bethyl, Hamburg, Germany) antibody (1:8000 in 1% skimmed milk TBST) served as the primary antibody. The secondary antibody was a rabbit-anti-goat HRP-conjugated antibody (Dianova, Hamburg, Germany) (1:5000 in 1% skimmed milk TBST). The visualisation of the marker (visible imaging) and the chemiluminescence bands of the same blot was conducted with the Fusion SL system (Vilber Lourmat, France).

### ELISA

The determination of IgM and IgG antibody titers followed a standard protocol [27]. Nunc-Immuno™ MicroWell™ 96 well solid plates (Sigma-Aldrich, Taufkirchen, Germany) were coated with 0.2% formaldehyde-inactivated bacteria (*S. suis cps7* strain 13-00283-02, *cps9* strain 16085/3b and cps2 strain 10cpsΔEF). Serum obtained from a *cps2* bacterin-vaccinated piglet (#4515) was used as reference serum and defined to include 100 ELISA units. Serum #4515 mediates killing of *S. suis* strain 10 (*cps*2), strain 13-00283-02 (*cps*7) and to a lesser extent also killing of strain 16085/3b (*cps*9) with bacterial survival factors in opsonophagocytosis assays at least tenfold (*cps*2 and *cps*7) or fourfold (*cps*9) lower than in the presence of serum from a colostrum-deprived piglet (results not shown). Convalescent sera, obtained from a piglet experimentally infected with a *S. suis cps9* strain, served as positive control and sera from colostrum-deprived piglets as negative control. Plates were washed three times with phosphate-buffered saline containing 0.05% Tween 20 (PBST) between incubation with antibodies and substrate. Serum from piglets of different age (4.5, 5.5, 6.5, 7.5, 8.5 and 10.5 weeks of life) was added as samples. For detection of serum IgM and IgG, polyclonal secondary goat-anti-porcine-IgM horseradish-peroxidase (HRP) conjugated antibodies were used, goat-anti-porcine-IgM (Thermo Scientific, Schwerte, Germany, 1:10 000 in PBST) and goat-anti-pig-IgG (A100-105P, Bethyl, Hamburg, Germany, 1:10 000 in PBST), respectively. Plates were developed using 2,2-azino-di-(3-ethylbenzithiazoline sulfonate) (ABTS, Roche, Mannheim, Germany) and H_2_O_2_ as the substrate. Absorbance was measured at 405 nm.

For absorption of sera with *S. suis* 10cpsΔEF, 2 mL of overnight cultures were centrifuged. Pelleted bacteria were resuspended in 200 μL of serum and rotated for 30 min at 4 °C. After centrifugation for 10 min at 10 000 × *g*, 100 μL of the supernatant was used as absorbed serum in ELISA.

### Bactericidal assay

Survival of bacteria was investigated in blood drawn weekly from 5 piglets aged 4.5–8.5 weeks from a herd classified as free of *cps7*+ and *cps9*+ strains. This classification was based on the multiplex PCR typing results [19] of *S. suis* isolates from the tonsils of more than 400 animals over the last 14 years [in the following this herd is referred to as specific pathogen free (spf)]. Furthermore, bacterial survival was investigated in blood drawn from 9 piglets every 2 weeks from a herd known to be infected with several *S. suis* serotypes, including *cps2*, *cps7* and *cps9*. The collection of blood samples was approved by the Landesdirektion Sachsen (permit nos. N01/16 and N19/14, respectively). The assay was performed essentially as described [20] but with 6 × 10^5^ CFU of exponentially grown *S. suis cps7* strains #451, V2310/1, 13-00283-02, D14412/3 and *S. suis cps9* strain 16085/3b mixed with 500 µL heparinized blood. CFU were determined on blood agar plates at t = 0 and t = 120 min with 2 h incubation on a rotator at 37 °C. In another approach, not only bacteria but also 10 µg recombinant Ide_*Ssuis*_ was added to examine the impact of IgM on bacterial survival in porcine blood of piglets at a certain age. The survival factor represented the ratio of CFU at 120 min to CFU at time zero.

### Animal experiments

Five spf landrace piglets were infected intravenously with 2 × 10^8^ CFU of *S. suis cps7* strain 13-00283-02 grown in Bacto™ Tryptic Soy Broth without dextrose (BD) and afterwards monitored every 8 h. In case of acute clinical signs such as polyarthritis, central nervous system dysfunction (e.g. convulsions) or in case of persisting high fever (≥ 40.5 °C) combined with anorexia and apathy euthanasia was carried out for animal welfare reasons. All piglets underwent necropsy, histopathological and bacteriological screenings as described previously [28]. The bacteriological screenings included analysis of α-hemolytic streptococci in the described MP-PCR. Isolates with a *mrp*+ *arc*A+ *gdh*+ *cps*7+ genotype were regarded as isolates of the challenge strain.

Weaning of all piglets included in this study was conducted in the 4th week of life.

### Statistical analysis

The evaluation of ELISA and bactericidal assays with more than two repeated measures was carried out using one- or two-way analysis of variance (ANOVA) with subsequent Dunn’s or Tukey’s multiple comparison tests. For comparison of ELISA data from one or two time points, the Shapiro–Wilk normality test and subsequently the *t* test were used. Means and standard deviation of the results are shown. Probabilities lower than 0.05 were considered significant (*p < 0.05, **p < 0.01, ***p < 0.001 and ****p < 0.0001).

## Results

### Invasive *S. suis cps7* strains recently isolated in Germany mainly belong to sequence type 29

**Figure 1:**
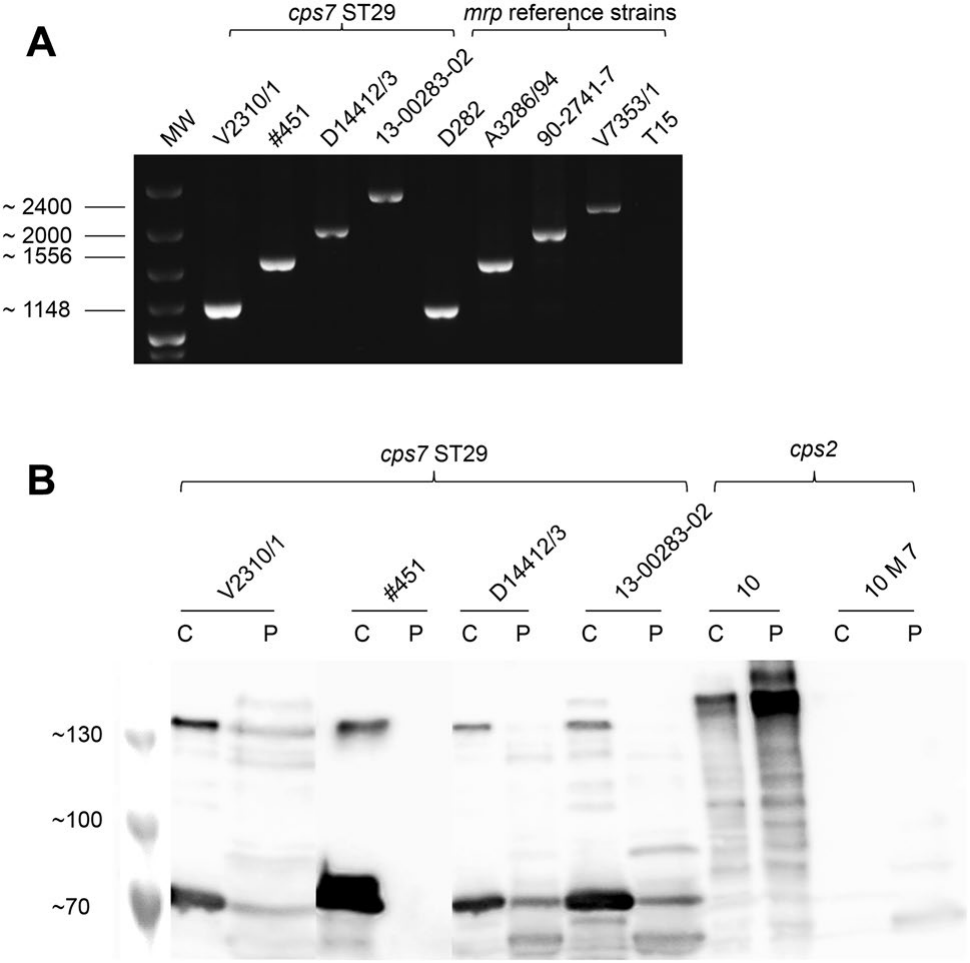
**PCR for differentiation of** ***mrp*** **variants (A) and detection of MRP variants by anti-MRP Western blot (B) in the indicated** ***S. suis*** **strains.** V2310/1 (*mrp*); #451 (mrp*); D14412/3 (mrp***); 13-00283-02 (mrp****); *mrp* reference strains: D282 (*mrp*); A3286/94 (mrp*); 90-2741-7 (mrp***); V7353/1 (mrp****); T15 (*mrp* negative reference strain). Strain 10 and the isogenic *mrp*-mutant 10M7 [24] served as MRP references in the Western blot. MW, 100 bp plus ladder (Invitrogen). Sizes of the PCR products (base pairs) and of the MRP variants (kDa) are indicated on the left. The Western blot was conducted using culture supernatant (C) and protoplast supernatant (P) as samples.

Different diagnostic laboratories and swine veterinarians reported an increased detection of *S. suis cps7* infections in association with severe herd problems in Germany and Austria between 2009 and 2016. Based on this observation, we hypothesized the emergence of a new clonal complex. Thus, 22 recent isolates of *S. suis cps7* were chosen for further analysis (Table 1). Most strains (12/22) were isolated from the brains of animals from herds with increased mortality of more than 5% at an age between 4 and 10 weeks, mainly due to meningitis. Seven originated from organs such as lung, spleen and joints from diseased piglets of affected herds and 2 from the tonsils of healthy carrier pigs. By MLST analysis 18 of the 22 isolates were shown to be ST29 strains, whereas the other four, including the two isolates from the tonsils, belonged to different STs (Table 1). Typing with a multiplex PCR for virulence-associated factors revealed that 19 of the 22 *cps7*+ strains generated an *mrp* amplification product, whereas only one strain was positive for the suilysin gene *sly*. Notably, four out of six described *mrp* variants were detected in these strains using an *mrp* variant PCR, with *mrp* generating the 1148 bp band, being the most frequent one (Figure 1A, Table 1). In conclusion, the majority of invasive *cps7* isolates in Germany belongs to ST29 and has an *mrp*+ *sly*− genotype.

### Invasive *S. suis cps7* ST29 strains express a truncated MRP

Culture and protoplast supernatants of the *cps7* strains were investigated in anti MRP Western blot analyses to detect released and surface-bound MRP, respectively (Figure 1B). Although different variants of the *mrp* gene were found (Figure 1A), only one pattern of bands was detected, irrespective of the *mrp* gene variant. This band pattern consisted of one band of approximately ~76 kDa and a further band at ~150 kDa. As shown in Figure 1B, MRP was mainly detected in the culture supernatant and only comparatively weak bands were found in the protoplast supernatant. Based on the band pattern, we hypothesized that the *cps7* strains express a truncated MRP due to a premature stop codon, as has been described for 3 American *cps7* isolates previously [16]. Four *S. suis cps7* strains (#451, V2310/1, 13-00283-02, D14412/3) were chosen for sequence analysis of the *mrp* gene to investigate this further. The *mrp* genes of these four strains all had a stop codon (TAA) at the same position as described by Fittipaldi et al. [16] for 3 North American *cps7* strains. Our sequences showed 100% homology to the coding sequence of the North American *cps7* strains. In agreement with Western blot results, the truncated MRP had a theoretical molecular weight of 76 kDa and was designated MRP^s^ in agreement with previous publications [12]. Thus, our results indicate that invasive German *cps7* ST29 strains secrete a truncated MRP protein (MRP^s^) in accordance with a premature stop codon.

### Expression of functional Ide_*Ssuis*_ is variable in the four investigated, invasive *S. suis cps7* ST29 strains

**Figure 2:**
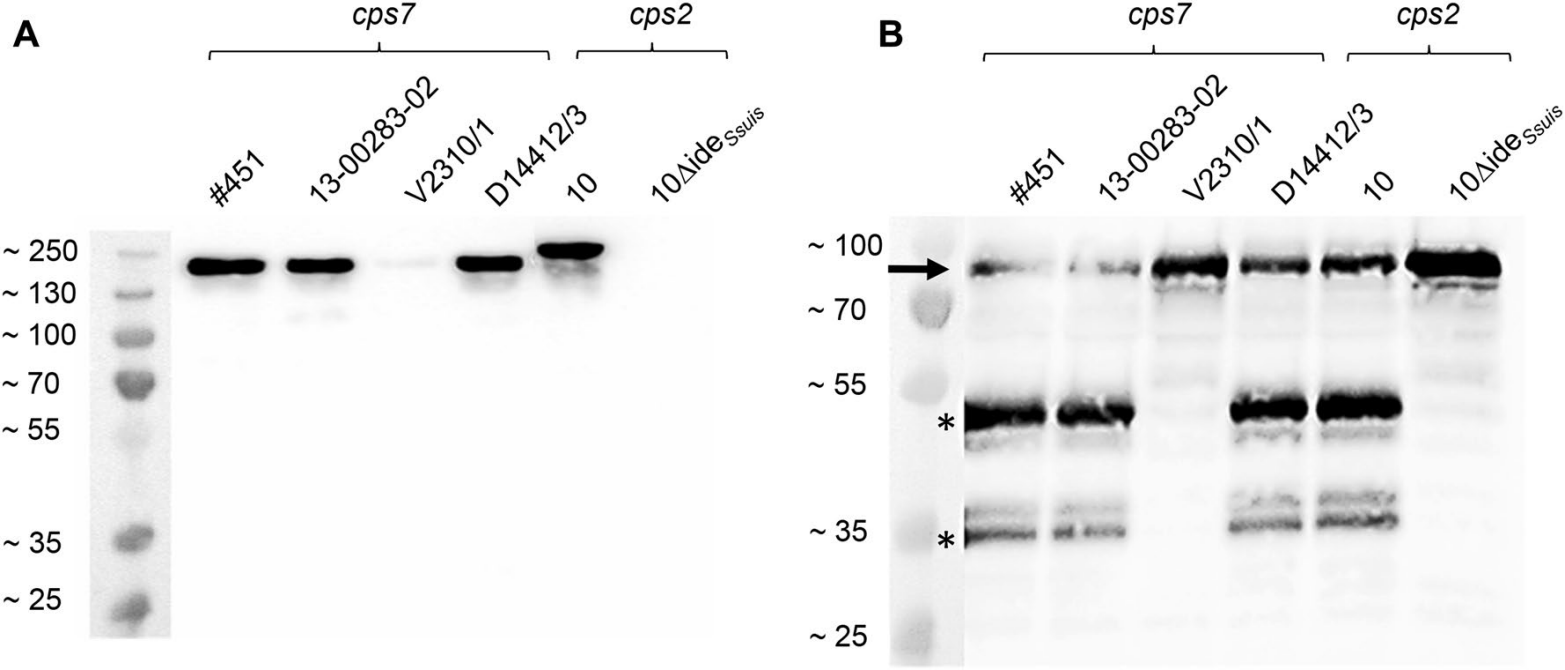
**Detection of Ide**_***Ssuis***_ **(A) and IgM cleavage products (B) in Western blot analysis. A** Anti-Ide_*Ssuis*_ Western blot of 30-fold concentrated culture supernatants of the indicated *S. suis* strains. **B** Anti-IgM Western blot of porcine serum incubated with 30-fold concentrated *S. suis* culture supernatants. The arrow marks the uncleaved IgM heavy chain, asterisks indicate cleavage products of the heavy chain at ~41 and ~32 kDa. The marker bands are shown on the left (sizes in kDa). Western blots were conducted under reducing conditions.

Ide_*Ssuis*_ is a highly specific IgM protease which was found to be expressed by all investigated *S. suis* strains in a previous study which also included one *cps7* strain, belonging, however, to ST27 [20]. Thus, it was not clear, if the emerging invasive *cps7* ST29 strains express functional Ide_*Ssuis*_. To clarify this, Western blot analyses for detection of Ide_*Ssuis*_ and IgM cleavage products were conducted. The anti Ide_*Ssuis*_ Western blot of the supernatants of the four selected *cps7* ST29 strains revealed specific bands between the 130 and the 250 kDa marker bands, showing small differences in size (Figure 2A). Strain V2310/1 showed a very weak Ide_*Ssuis*_ band. Noteworthy, PCR revealed that all four *cps7* strains carried the gene encoding Ide_*Ssuis*_ (Additional file 2). For functional analysis of Ide_*Ssuis*_, porcine serum was incubated with 30-fold concentrated culture supernatants of the *cps7* strains and analysed in an anti-IgM Western blot for detection of cleavage products (Figure 2B). In three of the four investigated *cps7* ST29 strains cleavage products with bands running at ~41 and ~32 kDa were detected. Noteworthy, the culture supernatant of strain V2310/1 did not show any IgM cleaving activity. In summary, expression of functional IgM protease Ide_*Ssuis*_ exhibits differences among *cps7* ST29 strains.

### *S. suis cps7* ST29 strains show high proliferation in blood of weaning piglets but an IgM-mediated killing in growing piglets of an infected herd

**Figure 3:**
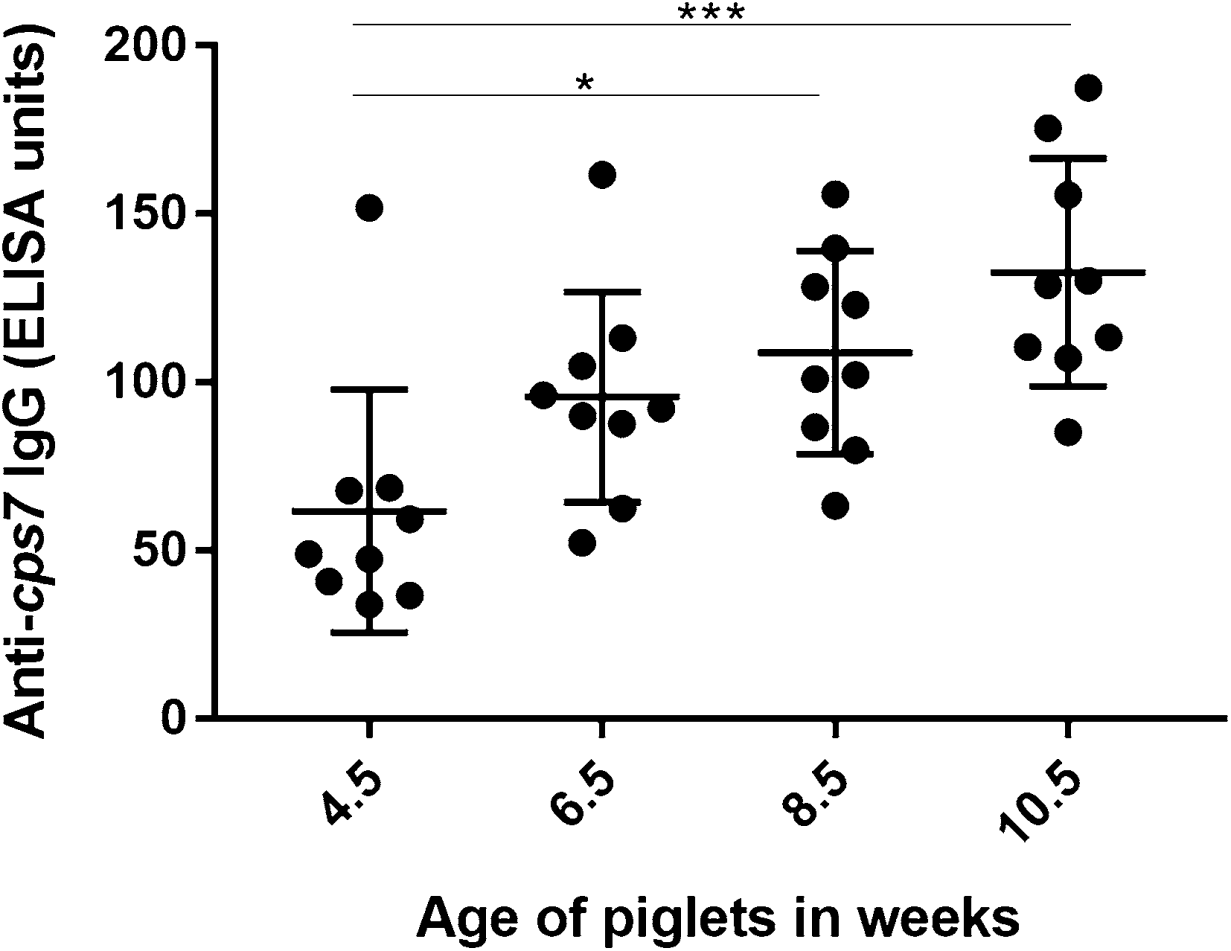
**Serum IgG antibody titers against** ***S. suis cps*7 ST29 strain 13-00283-02 increase between 4.5 and 10.5** **weeks of age in 9 randomly selected, healthy piglets from a herd known to be infected with** ***S. suis cps*7,** ***cps*2 and** ***cps*9.** IgG antibody titers of the 9 piglets were determined in sera every 2 weeks between 4.5 and 10.5 weeks of life. Titers rose significantly from 4.5 to 8.5 and 10.5 weeks of age. Means and standard deviations are indicated by horizontal lines and error bars, respectively. Significant differences were determined using one-way ANOVA with a consecutive Dunn’s multiple comparisons test. Significances are indicated (*p < 0.05, **p < 0.01, ***p < 0.001, ****p < 0.0001).

**Figure 4:**
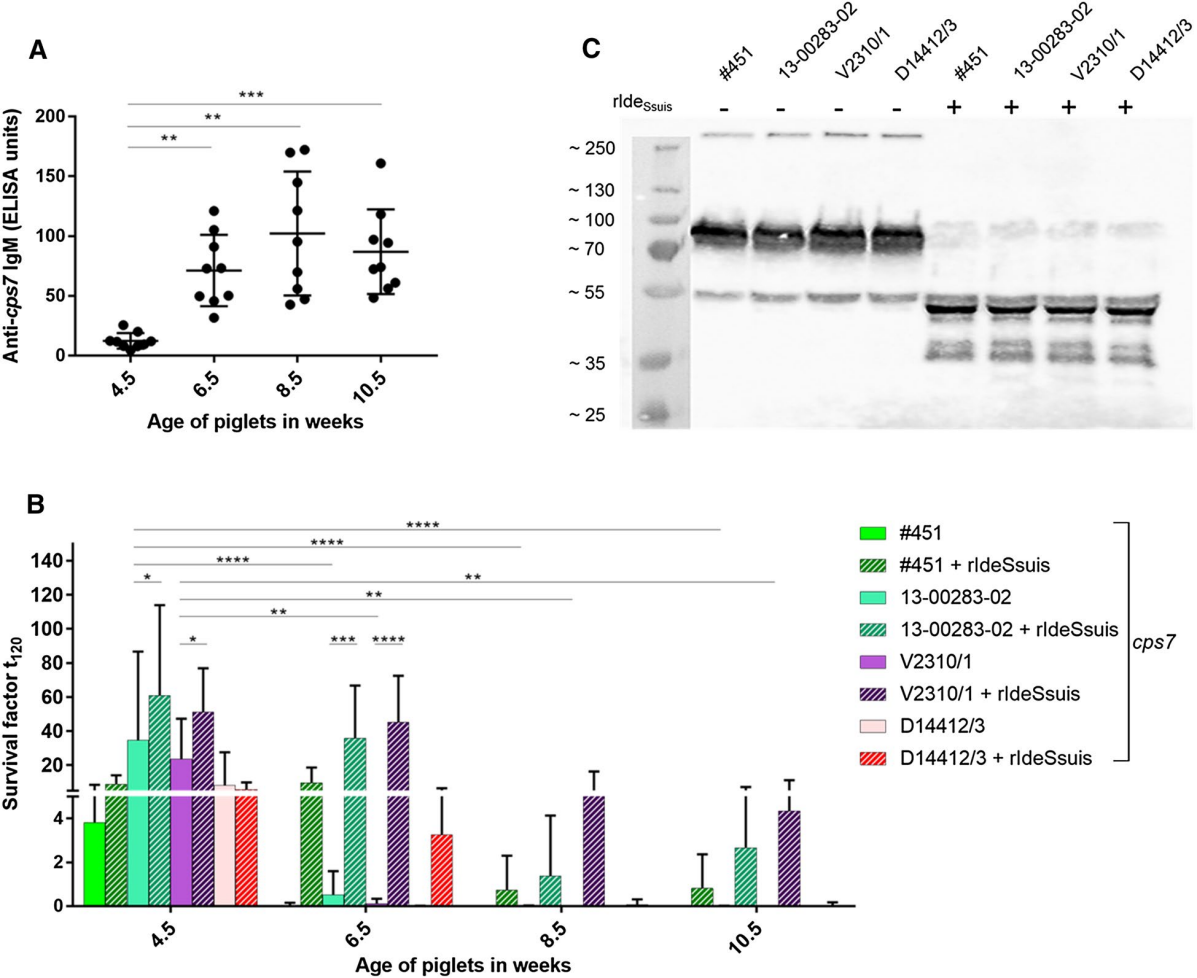
**Survival of** ***S. suis cps7*** **strains in the blood of 6.5** **week old piglets of a herd infected with different** ***S. suis*** **serotypes, including** ***cps7*, is partially IgM restricted. A** IgM titers against inactivated *S. suis cps7* (strain 13-00283-02) rose significantly in these piglets from 4.5 to 6.5 and 8.5 weeks of age. Horizontal lines and error bars indicate means and standard deviations, respectively. **B** Survival of the indicated *S. suis cps7* strains in porcine blood ex vivo is higher in weaning (4.5 weeks) than in growing piglets (8.5 and 10.5 weeks). Survival of the *S. suis cps7* strains was determined in blood drawn from the same piglets every 2 weeks. The survival factor represented the ratio of CFU at 120 min to CFU at time zero. To investigate the impact of intact IgM on bacterial survival, blood was incubated with the highly specific IgM protease rIde_*Ssuis*_ (10 µg). Bars and error bars represent mean values and standard deviations, respectively. **C** Anti-IgM Western blot for detection of IgM and its cleavage products in plasma samples of a piglet aged 4.5 weeks after bactericidal assays conducted as shown in **B**. Bactericidal assays included 10 μg rIde_*Ssuis*_ as indicated. Sizes of the marker bands (kDa) are indicated on the left. Significant differences were determined using one-way (**A**) or two-way ANOVA (**B**) and subsequently Tukey’s multiple comparisons test. Significances are indicated (*p < 0.05, **p < 0.01, ***p < 0.001, ****p < 0.0001).

Bacteremia is thought to be a crucial step in the pathogenesis of *S. suis* meningitis [29]. We asked if survival of *S. suis cps7* strains in the blood of piglets from an infected herd displays age-related phenotypes in relation to the putative changes of specific immunoglobulin titers after weaning. Thus, specific antibody titers and bacterial survival in blood were determined at different time points after weaning in a herd infected with numerous serotypes including *cps7*. At an age of 4.5 weeks, 9 randomly selected healthy piglets showed mean anti *S. suis cps7* IgG titers of 62 ELISA units (SD = 36) (Figure 3). Until 10.5 weeks of age an increase of specific IgG titers was observed with significant differences between 4.5 and 8.5 weeks as well as 10.5 weeks with mean titers of 109 (SD = 30) and 133 ELISA units (SD = 34), respectively (Figure 3). IgM titers against a *cps7* strain were very low with a mean titer of 12 ELISA units (SD = 7) at the time of weaning (4.5 weeks) but rose significantly from 4.5 to 6.5 weeks (Figure 4A). At least until the 10.5 week of age these IgM titers remained high with values above 40 ELISA units. Noteworthy, the increase in IgM (and IgG) was observed in all investigated piglets though none of the piglets displayed clinical signs of a disease related to *S. suis* infection.

Bactericidal assays were conducted at the described time points of serum sampling and addition of rIde_*Ssuis*_ was used as a tool to investigate the impact of IgM on bacterial survival. The four investigated *S. suis cps7* ST29 strains, which were randomly selected from our collection of invasive *cps*7 ST29 strains, showed very high survival factors in the blood of 4.5 week old piglets with mean survival factors between 4 and 35, indicating proliferation of the streptococci. The strains were efficiently killed in the blood of the same piglets 2 weeks later (Figure 4B), at a time when the piglets had just undergone a pronounced increase of specific IgM (Figure 4A). Addition of rIde_*Ssuis*_, a highly specific protease cleaving porcine IgM but not IgG [20], to the bactericidal assays of the 6.5 week old piglets in amounts sufficient to lead to complete cleavage of IgM (Figure 4C), resulted in a pronounced increase in survival of the *cps7* strains. This increase in bacterial survival through addition of rIde_*Ssuis*_ was statistically significant in 2 of 4 investigated strains and also seen in 8.5 and 10.5 week old piglets, but the effect was smaller in these older piglets. In conclusion, *S. suis cps7* ST29 showed high proliferation in the blood of weaning piglets with low specific IgM titers but was restricted very much in survival as IgM increased in these piglets.

### The survival pattern of *S. suis cps7* ST29 strains in porcine blood is distinct from other strains such as *cps9*

**Figure 5:**
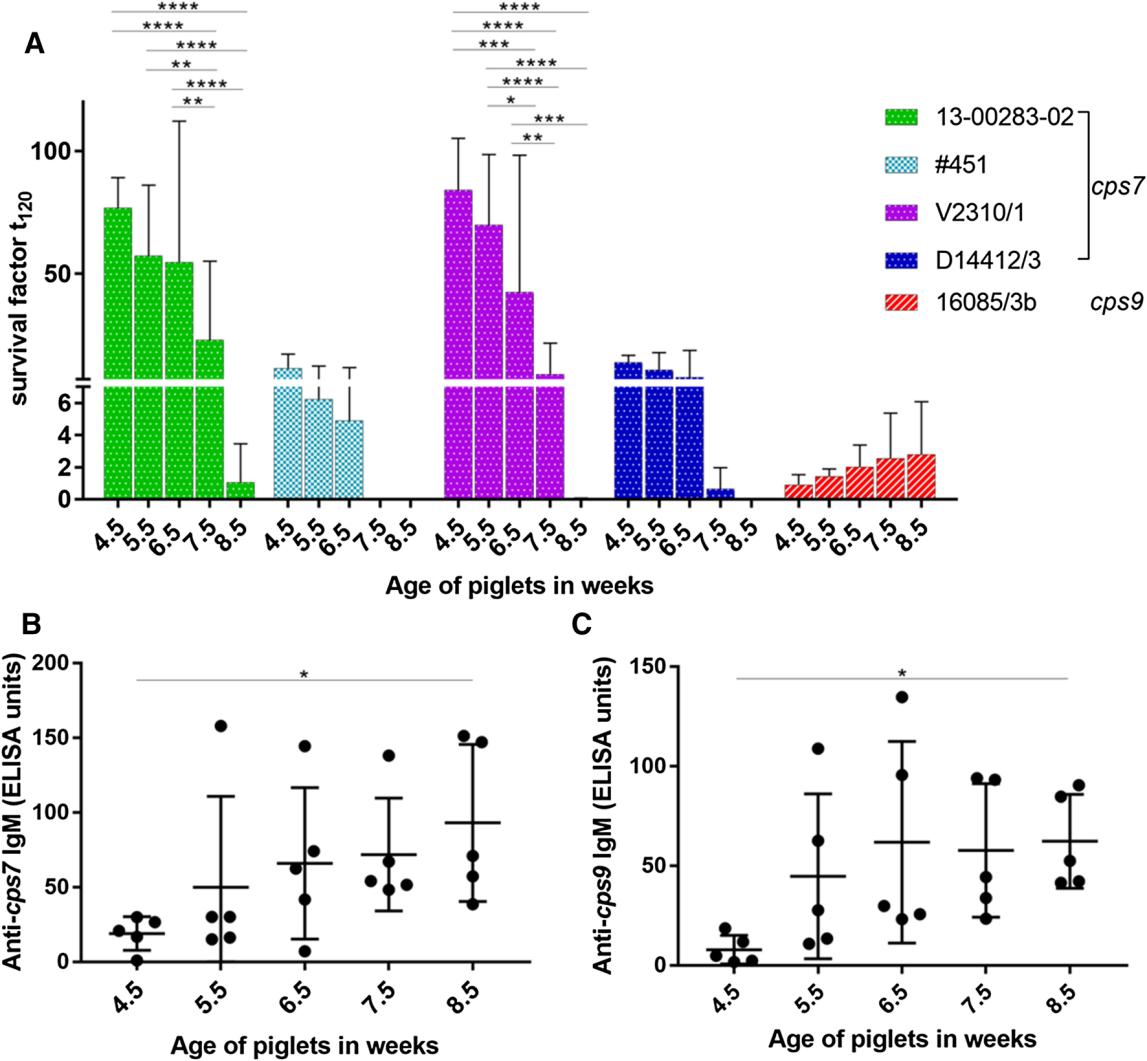
**Comparative analysis of survival of different** ***S. suis cps7*** **strains and a** ***cps9*** **strain in blood of weaning and growing piglets ex vivo (A) and IgM-antibody titers of these piglets against** ***cps7*** **(B) and** ***cps9*** **(C). A** Survival of *S. suis* strains was determined in porcine blood drawn from 5 piglets from a herd known to be free of *S. suis cps7* and *cps9* strains every week. Survival of *S. suis cps7* strains 13-00283-02 and V2310/1 is significantly higher in weaning (4.5–6.5 weeks) than in growing piglets (7.5 and 8.5 weeks). The survival factor represented the ratio of CFU at 120 min to CFU at time zero. Bars and error bars represent mean values and standard deviations, respectively. IgM titers against inactivated *cps7 S. suis* strain 13-00283-02 (**B**) and *cps9 S. suis* strain 16085/3b (**C**) were measured in the same blood samples as used for the bactericidal assays. IgM titers rose significantly from weaned to 8.5 weeks-old piglets. Means and standard deviations are indicated by horizontal lines and error bars, respectively. Significant differences were determined using two-way ANOVA and subsequently Tukey’s multiple comparisons test (**A**) and one-way ANOVA with Dunn’s (**B**) or Tukey’s (**C**) multiple comparisons test respectively. Significances are indicated (*p < 0.05, **p < 0.01, ***p < 0.001, ****p < 0.0001).

Next, we investigated the survival pattern of *S. suis cps7* ST29 strains in the blood of piglets from a herd known to be free of *S. suis cps7* and *cps9* strains to find out if the described IgM-mediated killing observed in growing piglets is related to *cps7 S. suis* infection and not induced by infection with other serotypes. As shown in Figure 5A, survival of *S. suis cps7* also decreased as weaning spf piglets became older. In this case, killing of the bacteria, which is indicated by survival factors smaller 1, set in at 8.5 weeks of age, 2 weeks later compared to piglets from the infected herd. In contrast to *cps7*, we observed only marginal changes in bacterial survival of a *cps9* strain in the blood drawn from the same spf weaning and growing piglets as these animals grew older. In contrast to *cps*7 ST29, the bacterial survival factor of the *cps9* strain was above 1 in the blood of 8.5 week old piglets.

Again, IgM antibodies binding to *S. suis cps7* were measured in serum drawn at the same time points as the assays were conducted. While IgM titers against *S. suis cps7* were hardly detectable in piglets aged 4.5 weeks, they rose notably with a significant difference between 4.5 and 8.5 weeks (Figure 5B). Significant differences between the *cps7*-infected and spf piglets in IgM titers against *S. suis cps7* strain 13-00283-02 at 4.5, 6.5 and 8.5 weeks of age were not detected (*p* = 0.2824, *p* = 0.8383, *p* = 0.7614, respectively). IgM titers were also measured against *S. suis cps9* strain 16085/3b and similar results were obtained. Piglets aged 4.5 weeks had rather low IgM titers, which then increased with a significant difference between 4.5 and 8.5 weeks (Figure 5C). Thus, the high proliferation in blood of weaning piglets as seen for *cps7* ST29 strains, could not be detected for a *cps9* strain, though IgM titers against both *S. suis* serotypes were low at an age of 4.5 weeks. As the piglets of this herd were regarded to be free of *cps*7 and *cps*9 strains, we figured that the adaptive IgM response should include IgM antibodies directed against other surface antigens than the capsular polysaccharides. As an unencapsulated mutant of *S. suis* ST29 was not available, we used the unencapsulated mutant of *cps*2 strain 10, namely 10cpsΔEF, to investigate the observed IgM response further. As shown in Additional file 3, absorption of sera with 10cpsΔEF resulted in a substantial reduction of antibodies at 6.5 weeks of age, detectable in the ELISA measuring antibodies against *S. suis cps*7 strain 13-00283-02. Furthermore, between 4.5 and 6.5 week of age spf piglets demonstrated a substantial increase in IgM antibodies binding to immobilised strain 10cpsΔEF (Additional file 4). These results suggested that the adaptive IgM immune response observed in these piglets included cross-reacting antibodies directed against other bacterial surface antigens than the capsular polysaccharides.

### Experimental infection of weaning piglets with *S. suis cps7* ST29 leads to severe clinical signs and pathologies

The bactericidal assays revealed that the *cps7* ST29 strain 13-00283-02 showed significantly higher survival factors in the blood of weaning piglets (4.5 weeks of age) than the *cps7* ST29 strains #451 and D14412/3, independently of the infection status of the piglets (*p* < 0.0001 in case of the spf piglets, *p* = 0.003 and *p* = 0.0224, respectively, in case of the piglets from the herd known to be infected with several *S. suis* serotypes). Differences between strain V2310/1 and strains #451 and D14412/3 were also notable. This was significant in case of the spf piglets (*p* < 0.0001).

Thus, we chose strain 13-00283-02 to confirm virulence in experimental infection and to establish an animal model for future vaccination and pathogenesis studies.

All five spf piglets infected intravenously with *S. suis cps7* strain 13-00283-02 at an age of 5 weeks demonstrated clinical signs in relation to polyarthritis (lameness, swollen joints, pain vocalization) and central nervous system dysfunctions (opisthotonus, ataxia, generalized tremor) within 36 h post-infection and were euthanized for animal welfare reasons. Histopathological screenings revealed severe lesions leading to a high score of ω = 4.4 (ω is calculated by division of the sum of the highest scores of each animal for any of the investigated organs by the number of animals: ω = Σscore_max_/*n*_animals_ [28]; 5 is the highest possible score). Severe, diffuse fibrinosuppurative meningitis was diagnosed in three piglets, whereas one piglet had a mild, diffuse mixed cell (neutrophils, mononuclear cells) meningitis. Furthermore, severe fibrinosuppurative polyarthritis was found in one piglet and mild multifocal mixed cell (neutrophils, mononuclear cells) serositis in all but one animal (Additional file 5). The infection strain was isolated from different organs of every piglet including cerebrospinal fluid and the brain. In conclusion, experimental infection confirmed that *S. suis cps7* ST29 is a virulent pathotype causing meningitis in weaning piglets.

## Discussion

Previous research on *cps* distribution of *S. suis* revealed the highest prevalences for *cps*9 and *cps*2, followed by other *cps* types such as *cps*1 and *cps7* in Europe [12, 19]. Invasive *cps2* strains mainly belong to ST1 but might also occur in ST25 and 28. ST16 is also important in Europe and associated with invasive isolates, mainly belonging to *cps9* [6, 11–13, 30]. Recently, *cps7* strains were detected in a number of cases with severe herd problems in Germany. All but two of our investigated invasive *cps7* strains belong to ST29 suggesting that ST29 *cps7* strains are emerging in Europe. Schultsz et al. describe a clonal complex 25 with ST29 as secondary founder based on the analysis of isolates obtained in the Netherlands [11], whilst ST29 is the primary founder of clonal complex 29 in the recent analysis of isolates from around the world conducted by Goyette-Desjardins et al. [6]. *Cps7* is dominating in this clonal complex [6], which is supported by the MLST database and our results.

In serotypes 2 and 9 strains, MRP is a surface-associated protein anchored to the cell-wall through an LPXTG-motif [19, 31]. Recently, MRP has been shown to mediate binding of fibrinogen to the bacterial surface of *S. suis cps2* contributing to survival in human blood as well as to adhesion and traversal across human cerebral microvascular endothelial cells [17, 18]. In this work, we describe an emerging *S. suis cps7* pathotype in Germany that secretes a short MRP variant of 76 kDa lacking an LPXTG motif. The reason for the additional 150 kDa band visible in the culture supernatants is not known but it might represent a dimer band of MRP^s^. In case the detected STOP codon in the *mrp* gene of the *cps7* strains had been overread, an LPXTG motif would be present and a prominent band of approximately 150 kDa should have also been detectable in the protoplast supernatant as in the case of MRP of *S. suis cps*2, which is, however, not the case (nucleotides TTGCCAAATACTGGT encoding LPNTG are still present in the downstream sequence of *mrp*^S^, see FJ685526.1 in GenBank). Though we did not conduct loss-of-function experiments, the fact that MRP^S^ was mainly found in the supernatant (Figure 1B) suggests that the main function of MRP^s^ is not to recruit fibrinogen to the bacterial surface. However, the fibrinogen binding domain was mapped to amino acids 283–721 in *cps2* MRP [18] and amino acids 283–697 of this region are conserved with 62% identity in MRP^s^ suggesting that MRP^s^ might still bind fibrinogen. Though a recent study by Wang et al. indicates that the interaction of fibrinogen with MRP on the bacterial surface promotes the development of meningitis in *S.* *suis* serotype 2 [17], *S. suis* ST29 strains secreting MRP^s^ were isolated from numerous cases with meningitis herd problems and meningitis was experimentally induced in four of five piglets intravenously infected in this study with a *S. suis cps7* strain secreting MRP^s^. Anchorage of MRP to the cell wall was obviously not crucial for the evolution of this *S. suis* pathotype and its ability to cause meningitis. As MRP is a main immunogen of *S. suis* [27], secretion of MRP^s^ might constitute an evasion mechanism against opsonophagocytic killing mediated by anti-MRP antibodies detectable in many piglets in Europe. In *cps2*, expression of MRP is associated with strains of increased virulence in Europe [14]. Whether expression of MRP^s^ is also a virulence marker in *cps7*, warrants further investigation.

Natural as well as adaptive IgM has been demonstrated to be effective in protection against *S. pneumoniae* infection using transgenic mice [32]. Furthermore, experimental studies in mice indicate that IgM is crucial for protection against relapsing fever due to infection with *Borrelia* species and recurrent episodes of high bacteremia [33, 34]. In this study, we used bactericidal assays with addition of the highly specific IgM protease Ide_*Ssuis*_ to assess the role of adaptive IgM in the control of *S. suis* bacteremia in the natural host. Survival of *S. suis cps7* ST29 in blood drawn from 6- to 8-week-old piglets with adaptive IgM was very much restricted by IgM. Noteworthy, all randomly selected piglets showed a prominent increase of anti *S. suis* specific IgM from very low values at an age of 4.5 weeks to much higher values at an age of 6–8 weeks, suggesting that in the field many piglets go through an early adaptive immune response against this pathogen during this time of life even if no clinical signs are detectable. Serological data obtained using an unencapsulated mutant of a *cps*2 strain as antigen suggests that adaptive IgM against *S. suis* in naturally infected pigs is not only directed against capsular polysaccharides but against other surface antigens. These adaptive IgM antibodies are putatively cross-reacting with different *cps* types and explain why we recorded an increase in IgM antibodies binding to *S. suis cps*7 ST29 though the piglets were regarded as free of *cps*7.

Importantly, survival of *S. suis cps7* ST29 strains in porcine blood showed an opposing trend to this increase of IgM. Though specific IgG also increased from week 4 to 10 (Figure 3), the sharp decline in bacterial survival from 4.5 to 6.5 weeks of age was mainly IgM-mediated as addition of Ide_*Ssuis*_ to the blood of 6.5-week-old piglets resulted in survival factors comparable to the ones in blood of 4.5 week old piglets and Ide_*Ssuis*_ is known to cleave only IgM and not IgG [20]. The increase in bacterial survival through addition of Ide_*Ssuis*_ was lessened at an age of 8.5 and 10.5 weeks, probably due to further increasing, most likely opsonizing, specific IgG.

Ide_*Ssuis*_ has been described to be expressed by all investigated *S. suis* strains [20]. One *cps*7 strain (V2310/1) investigated here expressed comparatively small amounts of Ide_*Ssuis*_, not sufficient to allow detection of IgM cleavage products. Our results indicated that the expression of Ide_*Ssuis*_ by *S. suis*, at least in the case of *cps7* ST29, is not sufficient to evade IgM-mediated killing in porcine blood with high IgM titers. However, *S. suis cps9* did not show this trend as survival factors were similar at 8.5 and 4.5 weeks of age. Our results suggested that *S. suis cps7* ST29 infections but not *cps9* infections should occur more often in young weaning piglets up to 6/7 weeks rather than in older growing piglets. Years ago, this was also observed in Denmark where field isolates of *cps7* were mostly isolated from piglets under 3 weeks of age [35]. Wisselink et al. observed no age-related difference in susceptibility to *S. suis* serotypes 2, 7 and 9 [12]. However, serotype 1 strains were mostly isolated from 3-week-old piglets, indicating that there are differences between serotypes regarding susceptibility at different age classes. Unfortunately, the age of the piglets the investigated *cps*7 strains were originally isolated from, is not well documented. Future epidemiological studies are needed to document the prevalences of *cps*7 infections at different age classes.

In summary, this study shows that invasive *cps7* ST29 strains might secrete MRP^s^ and proliferate efficiently in blood of weaning piglets with low IgM titers in accordance with experimental induction of arthritis and meningitis at this age. This is important for understanding host–pathogen interaction and development of vaccines against this emerging pathotype.

## Additional files

**Additional file 1.** **Sequences of oligonucleotide primers.** Name, sequence and position of primer sequences used for *mrp* sequencing.

**Additional file 2.** **Detection of** ***ide***_***Ssuis***_ **in different** ***S. suis cps7*** **strains via PCR.** Primers that bind in the conserved region of *ide*_*Ssuis*_ (IdeSsuis_con_fo: GGGGAAGTAGCGGTAGAGATGAAAG and IdeSsuis_con_re: GATTGACACCGCCCTGTGCC) were used for amplification of *ide*_*Ssuis*_ in *cps7* strains #451, 13-00283-02, V2310/1 and D14412/3. Strain 10 served as positive and 10∆ide_*Ssuis*_ as negative reference strains. MW, 100 bp plus ladder (Invitrogen). Sizes of selected marker bands (in base pairs) are indicated on the left.

**Additional file 3.** **IgM titers in the sera of the investigated 6.5** **week old** ***cps*7 free piglets against** ***cps*7 strain 13-00283-02 are substantially reduced after preabsorption with strain 10cpsΔEF.** IgM antibody titers of the 5 piglets investigated for the data presented in Figure 5 were also determined after absorption of the sera with the unencapsulated *cps2* mutant strain 10cps∆EF. Means and standard deviations are indicated by horizontal lines and error bars, respectively. Each symbol represents a different animal. Differences were not significant using a two-tailed paired t-test.

**Additional file 4.** **Titers of serum IgM antibodies binding to the unencapsulated strain 10cpsΔEF increase in the five investigated** ***cps*7 free piglets from 4.5 to 6.5** **weeks of age.** The unencapsulated mutant 10cpsΔEF of *cps*2 strain 10 was used as antigen in an ELISA for determination of IgM antibodies binding to other *S. suis* antigens but capsular polysaccharides in the 5 piglets investigated for the data presented in Figure 5, which were from a herd considered free of *cps*7 and *cps*9. Means and standard deviations are indicated by horizontal lines and error bars, respectively. Each symbol represents a different animal. Differences were not significant using a two-tailed paired t-test.

**Additional file 5.** **Scoring of fibrinosuppurative lesions of piglets challenged with** ***S.suis cps7*** **strain 13-00283-02 (*mrp***+ ***cps7*).** Five spf piglets were infected intravenously with 2 × 10^8^ CFU of *S. suis cps7* ST29 strain 13-00283-02 and afterwards monitored every 8 h. All piglets demonstrated clinical signs in relation to polyarthritis (lameness, swollen joints, pain vocalization) and/or central nervous system dysfunctions (opisthotonus, ataxia, generalized tremor) within 36 h post-infection and were euthanized for animal welfare reasons. Necropsies and histopathological screenings of the indicated tissues were conducted with all 5 piglets as described [28].

## Abbreviations

*S.*: *Streptococcus*
ST: sequence type
MRP: muramidase-released protein
EF: extracellular factor protein
Ide_*Ssuis*_: immunoglobulin M-degrading enzyme of *Streptococcus suis*
Ig: immunglobulin
MLST: multi locus sequence typing
spf: specific pathogen free
