# Supplementary material for: Streptococcus suis cps7: an emerging virulent sequence type (ST29) shows a distinct, IgM-determined pattern of bacterial survival in blood of piglets during the early adaptive immune response after weaning
Source: Vet Res. 2018 Jun 15;49:48. doi: 10.1186/s13567-018-0544-8 (PMC6003162; doi:10.1186/s13567-018-0544-8)
Supplement: Supplementary file 1 — Additional file 1. Sequences of oligonucleotide primers. Name, sequence and position of primer sequences used for mrp sequencing. [file 13567_2018_544_MOESM1_ESM.doc]

| **Primer** | **Sequence** | **Position^a^** |
| --- | --- | --- |
|  |  |  |
| mrp7_1_for | AGTAGAAAATTTGTGTAATTAAATTAAT | -49 to -21 |
| mrp7_1_rev | CACCAACAATTGAACCATTCTGG | 785 to 808 |
| mrp7_2_for | GCTACAAAAGAATCTGGTTGG | 723 to 744 |
| mrp7_2_rev | TACCTCTCCATTGGTTTTCGG | 1702 to 1723 |
| mrp7_3_for | AAACCAGAGTATACCGGAGG | 1620 to 1640 |
| mrp7_3_rev | GGTGTCGTTGGATCGATTGG | 2708 to 2728 |
| mrp_rev | TGAGCTTTACCTGAAGCGGT | 4516 to 4536 |
| mrp_var_for | GACAGATGGTGAGGAAAATGG | 2551 to 2572 |
|  |  |  |

**Additional file 1 Sequences of oligonucleotide primers.** Name, sequence and position of primer sequences used for *mrp* sequencing.

^a^ Numbers mark the primer position with regard to the coding sequence (49-2172) of *mrp* (GenBank Accession No.: FJ685526.1)
