# Supplementary figures and images for: Streptococcus suis cps7: an emerging virulent sequence type (ST29) shows a distinct, IgM-determined pattern of bacterial survival in blood of piglets during the early adaptive immune response after weaning
Source: Vet Res. 2018 Jun 15;49:48. doi: 10.1186/s13567-018-0544-8 (PMC6003162; doi:10.1186/s13567-018-0544-8)

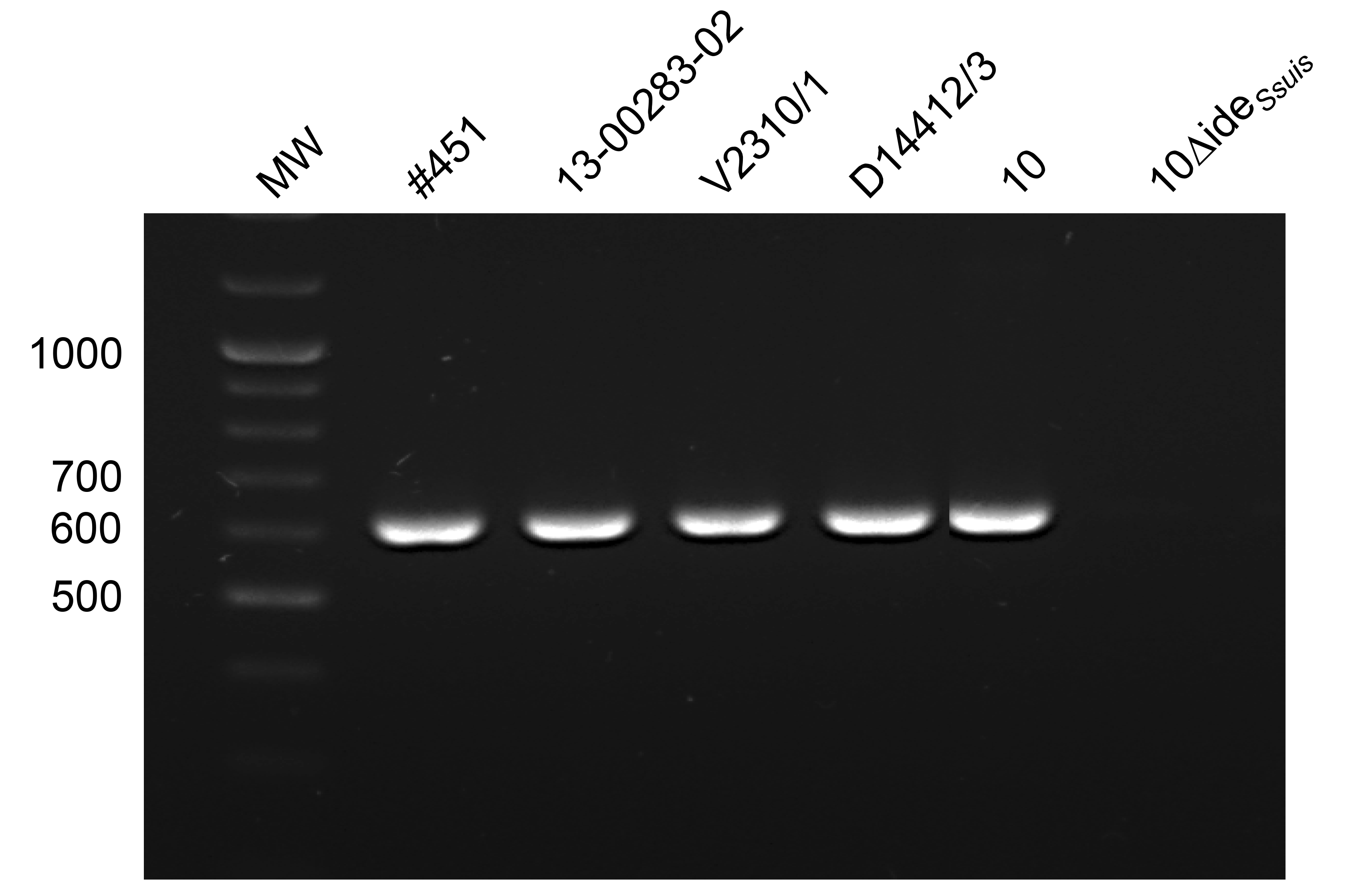

Supplement: Supplementary file 2 — Additional file 2. Detection of ideSsuis in different S. suis cps7 strains via PCR. Primers that bind in the conserved region of ideSsuis (IdeSsuis_con_fo: GGGGAAGTAGCGGTAGAGATGAAAG and IdeSsuis_con_re: GATTGACACCGCCCTGTGCC) were used for amplification of ideSsuis in cps7 strains #451, 13-00283-02, V2310/1 and D14412/3. Strain 10 served as positive and 10∆ideSsuis as negative reference strains. MW, 100 bp plus ladder (Invitrogen). Sizes of selected marker bands (in base pairs) are indicated on the left. [file 13567_2018_544_MOESM2_ESM.tif]

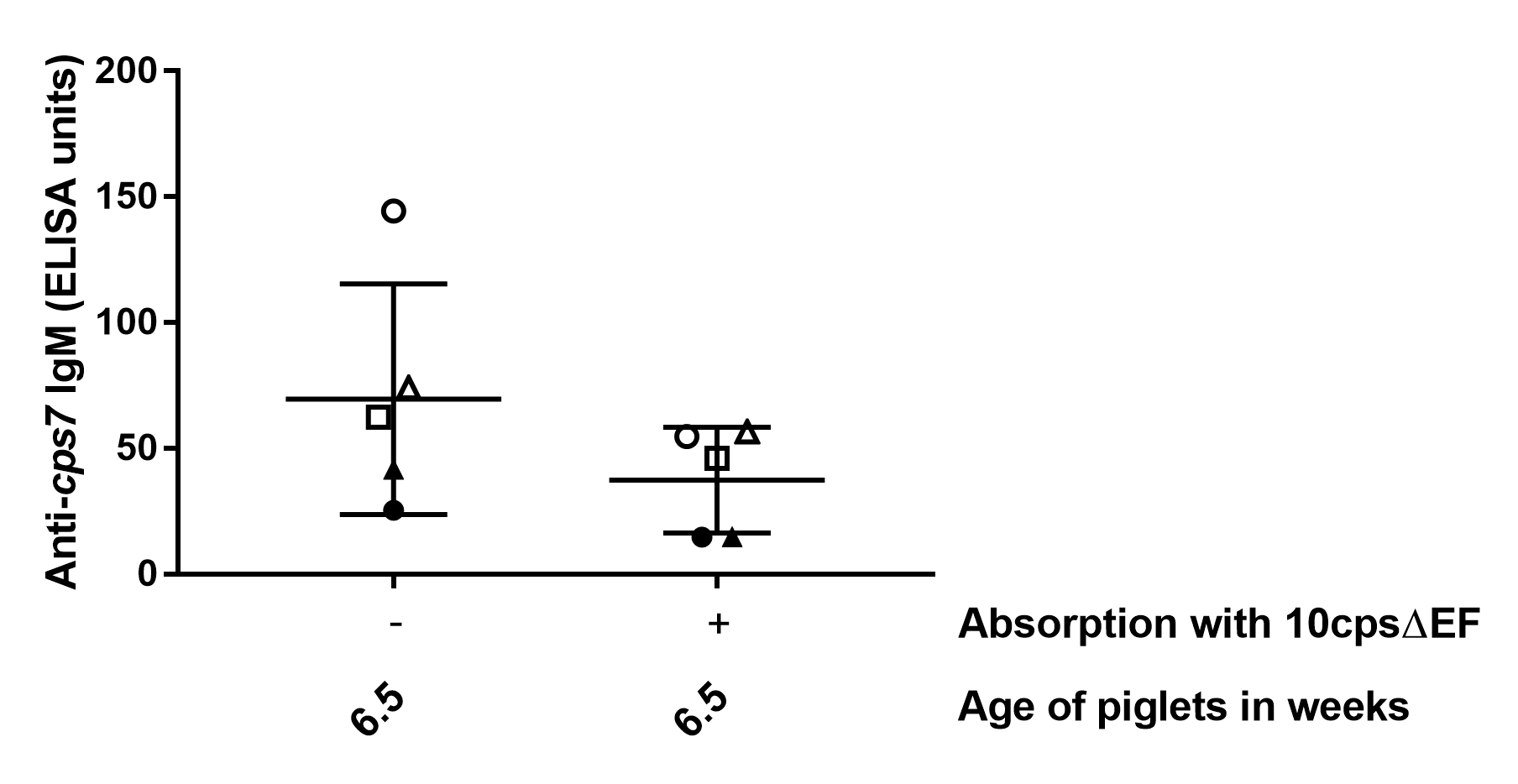

Supplement: Supplementary file 3 — Additional file 3. IgM titers in the sera of the investigated 6.5 week old cps7 free piglets against cps7 strain 13-00283-02 are substantially reduced after preabsorption with strain 10cpsΔEF. IgM antibody titers of the 5 piglets investigated for the data presented in Figure 5 were also determined after absorption of the sera with the unencapsulated cps2 mutant strain 10cps∆EF. Means and standard deviations are indicated by horizontal lines and error bars, respectively. Each symbol represents a different animal. Differences were not significant using a two-tailed paired t-test. [file 13567_2018_544_MOESM3_ESM.tif]

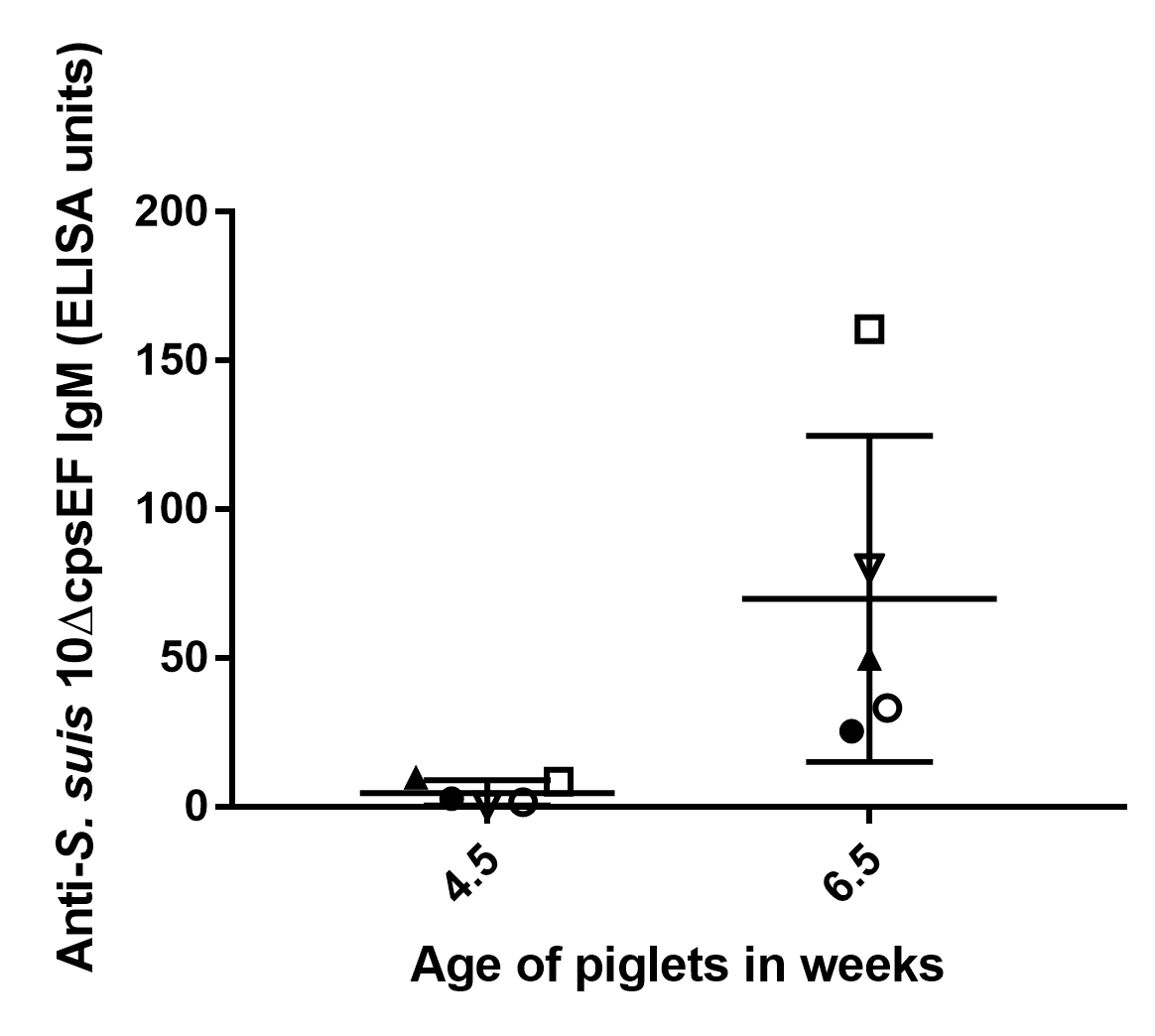

Supplement: Supplementary file 4 — Additional file 4. Titers of serum IgM antibodies binding to the unencapsulated strain 10cpsΔEF increase in the five investigated cps7 free piglets from 4.5 to 6.5 weeks of age. The unencapsulated mutant 10cpsΔEF of cps2 strain 10 was used as antigen in an ELISA for determination of IgM antibodies binding to other S. suis antigens but capsular polysaccharides in the 5 piglets investigated for the data presented in Figure 5, which were from a herd considered free of cps7 and cps9. Means and standard deviations are indicated by horizontal lines and error bars, respectively. Each symbol represents a different animal. Differences were not significant using a two-tailed paired t-test. [file 13567_2018_544_MOESM4_ESM.tif]
