# Supplementary material for: Streptococcus suis cps7: an emerging virulent sequence type (ST29) shows a distinct, IgM-determined pattern of bacterial survival in blood of piglets during the early adaptive immune response after weaning
Source: Vet Res. 2018 Jun 15;49:48. doi: 10.1186/s13567-018-0544-8 (PMC6003162; doi:10.1186/s13567-018-0544-8)
Supplement: Supplementary file 5 — Additional file 5. Scoring of fibrinosuppurative lesions of piglets challenged with S.suis cps7 strain 13-00283-02 (mrp+ cps7). Five spf piglets were infected intravenously with 2 × 10^8^ CFU of S. suis cps7 ST29 strain 13-00283-02 and afterwards monitored every 8 h. All piglets demonstrated clinical signs in relation to polyarthritis (lameness, swollen joints, pain vocalization) and/or central nervous system dysfunctions (opisthotonus, ataxia, generalized tremor) within 36 h post-infection and were euthanized for animal welfare reasons. Necropsies and histopathological screenings of the indicated tissues were conducted with all 5 piglets as described [28]. [file 13567_2018_544_MOESM5_ESM.doc]

**Additional file 5 Scoring of fibrinosuppurative lesions of piglets challenged with *S.suis cps 7* strain 13-00283-02 (*mrp+ cps7*).**

| Piglets without lesions^a^ | Piglets with lesions in two or more locations^a^ | Brain | | |  | Serosa | | |  | Joint | | |  | Spleen and liver | | |  | Lung | | |  | Heart | | |  | | |
| --- | --- | --- | --- | --- | --- | --- | --- | --- | --- | --- | --- | --- | --- | --- | --- | --- | --- | --- | --- | --- | --- | --- | --- | --- | --- | --- | --- |
|  |  | Meningitis, chorioiditis | | |  | Pleuritis or peritonitis or pericarditis | | |  | Synovialitis | | |  | Splenitis^b^ or hepatitis | | |  | Pneumonia | | |  | Endocarditis | | | |  |  |
|  |  | 5^c^ | 3^d^ | 1^e^ |  | 4^c^ | 2^d^ | 1^e^ |  | 4^c^ | 2^d^ | 1^e^ |  | 4^c^ | 2^d^ | 1^e^ |  | 4^c^ | 2^d^ | 1^e^ |  | 4^c^ | 2^d^ | 1^e^ | |  | ω^f^ |
| 0/5 | 5/5 | 3/5 | 1/5 | 1/5 |  | 0/5 | 4/5 | 1/5 |  | 1/5 | 3/5 | 0/5 |  | 3/5 | 2/5 | 0/5 |  | 4/5 | 1/5 | 0/5 |  | 0/5 | 0/5 | 3/5 | |  | **4.4** |

^a^ Only fibrinosuppurative lesions are considered. Individual single perivascular neutrophils are not counted.

^b^ Neutrophilic accumulation of the splenic red pulp.

^c^ Scoring of 4 and 5 indicates moderate to severe diffuse or multifocal fibrinosuppurative inflammations.

^d^ Scoring of 2 and 3 indicates mild focal fibrinosuppurative inflammation.

^e^ Individual single perivascular neutrophils received a score of 1.

^f^ ω = Σscore_max_/n_animals_
